# Multimodal neuroimaging reveals brain neurochemical disturbances associated with superoxide dismutase in first-episode drug-naïve schizophrenia

**DOI:** 10.1038/s41398-025-03801-w

**Published:** 2026-01-05

**Authors:** Zijia Zhu, Zhuo Wang, Xiuxia Yuan, Yawen Zou, Chenxiang Zheng, Yanqin Wen, Gangrui Hei, Xueqin Song, Yongyong Shi

**Affiliations:** 1 Bio-X Institutes, Key Laboratory for the Genetics of Developmental and Neuropsychiatric Disorders (Ministry of Education), Shanghai Jiao Tong University; Collaborative Innovation Centre for Brain Science, Shanghai Jiao Tong University, Shanghai, China; 2 Department of Psychiatry, the First Affiliated Hospital of Zhengzhou University; Henan International Joint Laboratory of Biological Psychiatry; Henan Psychiatric Transformation Research Key Laboratory/Zhengzhou University, Zhengzhou, China; 3 Institute of Neuroscience, State Key Laboratory of Neuroscience, Center for Excellence in Brain Science and Intelligence Technology (CEBSIT), Chinese Academy of Sciences, Shanghai, 200031 China

**Keywords:** Human behaviour, Schizophrenia

## Abstract

Reduced antioxidant defense observed in first-episode drug-naïve schizophrenia (SCZ) may contribute to impaired cognition of SCZ. However, the underlying relationships among antioxidant stress-neuroimaging-cognition pathway and neurotransmitter dysfunction remain unclear. In this study, 75 patients with first-episode drug-naïve SCZ and 85 age and sex matched healthy controls underwent clinical evaluation and brain MRI. Two brain imaging measurements, including gray matter volume (GMV) from structural MRI (sMRI) and fractional amplitude of low frequency fluctuations (fALFF) from resting-state functional MRI, were jointly analyzed with a data-driven multivariate fusion method. Serum superoxide dismutase (SOD) levels was used as a reference to guide fusion of two MRI features. To investigate specific neurotransmitter system alterations associated with SOD-related SCZ patients, we used the JuSpace toolbox for cross-modal correlations between two MRI-based modalities with nuclear imaging derived estimates. Two multimodal components (IC5 and IC7) show significant group difference in loadings. We found significant associations of SOD with both GMV and fALFF within the frontoparietal and default mode networks (DMN). Furthermore, the covariant structural-functional components linked with reduced SOD levels were associated with deficits in cognitive function in speed of processing and visual learning. Moreover, the spatial correlation analysis identified associations of altered neuroimaging fusion biomarkers with disrupted excitatory-inhibitory neurotransmitter receptor distribution. This study unveils the complex alterations in SCZ across structural, functional, and neurochemical dimensions through multimodal analysis, highlighting the association between SOD-induced oxidative stress and imbalances in the excitatory/inhibitory ratio in the core brain regions of the DMN. Antioxidant therapy may have the potential to alleviate cognition deficit in SCZ patients in the future.

## Introduction

Schizophrenia is a severe, debilitating and complex psychiatric disorder that affects approximately 1% of people worldwide [1]. Patients with schizophrenia often experience a range of symptoms including hallucinations, delusions, disorganized thinking, and altered behavior [2]. Neuroimaging research has identified specific brain abnormalities such as changes in the superior temporal gyrus, prefrontal cortex, and limbic system that correlate with cognitive impairments in schizophrenia patients [3, 4]. In fact, cognitive impairments, including deficits in learning, working memory, and social interaction contribute significantly to disability in schizophrenia [5]. In particular, brain regions associated with the default mode network (DMN) play a critical role in the clinical symptoms and cognitive impairments observed in patients with schizophrenia [6–8]. These results highlight a disrupted network of critical brain regions associated with cognitive function in schizophrenia, but the possible causes remain unclear.

Oxidative stress refers to an imbalance in the redox state of cells due to the excessive generation of free radicals and reactive oxygen species (ROS) [9]. This imbalance can lead to cellular damage and dysfunction, especially in the brain, which is highly susceptible to oxidative damage. Recently, oxidative stress has been identified as a central pathological mechanism in a range of neurological disorders, including schizophrenia [10–12]. Murray et al. [13] have described how oxidative stress may play a role in psychosis hypotheses involving dopamine, glutamate, the immune system, and impaired brain connectivity. An overabundance of ROS, often due to increased dopaminergic activity, can disrupt the normal function of dopamine transporters, culminating in increased synaptic dopamine concentrations and subsequent oxidative stress [13]. This interplay is further complicated by the potential for hypofunction of the NMDA receptor to induce excitotoxicity and augment ROS production, thereby perpetuating a cycle of neuronal damage [14]. The typically inhibitory GABAergic system can also be disturbed under conditions of oxidative stress, leading to an imbalance in neurotransmitter regulation and potentially exacerbating psychotic symptoms [15]. Early clinical studies reported that N-acetylcysteine (NAC) significantly improved the total PANSS score, negative symptoms, and clinical global impression score in patients with chronic schizophrenia, but was ineffective against positive symptoms, suggesting its potential as a safe adjunctive therapy [16]. Subsequent studies further confirmed that NAC has a broad effect on positive/negative/cognitive symptoms in chronic patients [17]. However, studies in patients with early psychosis have not verified the efficacy of NAC on negative symptoms, but it has been found to increase brain glutathione levels and improve neurocognitive function [18]. Despite the heterogeneity of treatment outcomes, intervention strategies targeting oxidative stress have important therapeutic value given the complexity of the clinical symptoms of schizophrenia. Moreover, a recent systematic review of cross-sectional studies (3002 first-episode psychosis patients and 2806 controls) showed that patients with first-episode psychosis have significantly lower total antioxidant status and docosahexaenoate levels, along with higher homocysteine, IL-6, and TNF-α levels compared to healthy controls [19]. These results emphasize the importance of exploring the complex relationship between oxidative stress and neurotransmitter dysregulation in the pathogenesis of schizophrenia.

Superoxide dismutase (SOD) is a very powerful antioxidant enzyme and can remove ROS from the cell. Numerous studies have found that SOD levels and activity are disrupted in individuals with schizophrenia and this disruption is associated with impaired cognitive function [20]. While oxidative stress can broadly impair cell viability, its impact on the brain is likely mediated through specific, sensitive pathways. As neurotransmitter systems are heterogeneously distributed and are key drivers of synaptic plasticity and neural communication, their dysregulation would directly alter synaptic efficacy and circuit function [21, 22]. Chronic dysregulation of synaptic plasticity is a core mechanism underpinning both gray matter loss and aberrant neural activity through dendritic spine pruning and altered neuropil [23, 24]. Therefore, we hypothesize that SOD-associated oxidative stress contributes to the structural and functional brain alterations observed in schizophrenia by disrupting the neurochemical substrates that maintain synaptic and circuit homeostasis.

To validate this hypothesis, we analyzed the covariance between peripheral SOD levels and various structural and functional features of the brain. Specifically, we performed a joint decomposition of two MRI features relative to individual peripheral SOD levels [25]: gray matter volume (GMV) from structural MRI (sMRI), fractional amplitude of low-frequency fluctuations (fALFF) from resting-state functional MRI (rs-fMRI). GMV captures the net effect of neuronal and glial cell volume, as well as the density of dendritic arbors and spines. Redox imbalance-induced impairments in neurotransmitter signaling may lead to synaptic loss and dendritic simplification, which are directly reflected in GMV measurements [26]. fALFF reflects the intensity of regional spontaneous neural activity, which is tightly coupled to underlying synaptic transmission and energy metabolism [27]. Alterations in neurotransmitter receptor function would directly perturb local neural dynamics, which fALFF is highly sensitive to [28, 29]. By fusing GMV and fALFF, we aim to identify brain patterns where the structural correlates of synaptic integrity and the functional correlates of local neural activity co-vary with SOD levels, providing a more integrated picture of oxidative stress impact. To better understand the relationship between SOD-related brain abnormalities and the underlying molecular features, we then performed cross-modal spatial correlation between positron emission tomography (PET) or single photon computed emission tomography (SPECT) derived neurotransmitters and the covariant structural-functional components linked with SOD levels through the JuSpace toolbox [22, 30]. PET and SPECT, using a variety of novel radioactive tracers, can reliably measure the availability of specific receptors as well as functional aspects such as synthesis capacity for different neurotransmitters, and provide a more direct measurement of specific tissue properties. JuSpace is an open source software package that contains various PET and SPECT-derived neurotransmitter receptor/transporter maps, including serotonin, dopamine, noradrenaline, and GABA (gamma-aminobutyric acid) neurotransmission, and allows for the integration of MRI phenotypes for specific brain conditions with existing maps of neurotransmitter binding and distribution [22, 30]. This approach provides insights into the association of these neuroimaging markers with SOD levels and contributes to elucidating the neurobiological mechanisms underlying schizophrenia. In particular, by examining the effects of oxidative stress and neuroimaging features on cognitive function, it may provide theoretical support for the development of early-diagnosis and targeted treatment strategies of SCZ patients.

## Materials and methods

### Participants

Seventy-five patients with SCZ and 85 HCs were recruited between October 2017 and January 2020 (Table 1). The inclusion criteria included (1) diagnosis of first-episode schizophrenia (FES) based on the criteria of the Diagnostic and Statistical Manual of Mental Disorders fourth version (DSM-IV) and confirmed using the Structured Clinical Interview for DSM-IV (SCID), (2) no prescription medication use and (3) a PANSS total score ≥ 60. All participants were examined by a psychiatrist. The exclusion criteria included (1) diagnosis of neurological diseases, diabetes, heart disease, digestive system diseases, autoimmune diseases, blood diseases or psychiatric diseases other than SCZ, (2) pregnancy or breastfeeding, (3) treatment with an antibiotic or anti-inflammatory agent in the last month and (4) obesity (BMI > 28 kg/m^2^). HCs were recruited from local communities through online advertisements and completed a comprehensive clinical evaluation, physical examination, and routine laboratory testing to rule out physical or mental illness. The PANSS scores included positive symptoms, negative symptoms, and general psychopathological subscales, represented by PANSS-P, PANSS-N, and PANSS-G, respectively. A higher PANSS score in schizophrenia indicates more severe clinical symptoms. Cognitive function was assessed using the MATRICS Consensus Cognitive Battery (MCCB) [5]. The raw assessment parameters corresponding to seven domains of cognitive function showed as following: (1) Speed of processing: Trail Making Test (TMT), Part B, Brief Assessment of Cognition in Schizophrenia-Symbol Coding (BACS-SC) and Category Fluency: Animal Naming (Fluency); (2) attention/vigilance: Continuous Performance Test-Identical Pairs (CPT-IP); (3) working memory: Wechsler Memory Scale-III Spatial Span (WMS-III SS); (4) verbal learning: Hopkins Verbal Learning Test-Revised; (5) visual learning: Brief Visual Memory Test-Revised; (6) reasoning and problem solving: Neuropsychological Assessment Battery (NAB) Mazes; and (7) social cognition: Mayer-Salovey-Caruso Emotional Intelligence Test (MSCEITTM, Managing Emotions). A lower MCCB score indicates a more severe cognitive impairment. All participants provided written informed consent. All methods were carried out in accordance with relevant guidelines and regulations. The present study was approved by the Human Ethics Committee of the First Affiliated Hospital of Zhengzhou University, China (Approval No. 2016-LW-17).

**Table 1:** Demographic and clinical information of subjects.

	SCZ	HC	*p* value (χ2 or
	n = 75	n = 85	Mann-Whitney U)
**TIV (mm**^**3**^**)**	1582 (161)	1601 (140)	0.442^a^
**Education (years)**	10.7 (2.90)	12.4 (1.37)	<0.001^a^
**Gender**			
Female	43 (57.3%)	53 (62.4%)	0.628^b^
**Age (years)**	22.3 (7.79)	23.0 (2.35)	0.509^a^
**Duration of illness (months)**	7.2 (8.7)		
**Smoking history (yes/total)**	4 (5.3%)	2 (2.4%)	0.322 ^b^
**PANSS**			
Positive	19.8 (4.25)		
Negative	20.8 (6.18)		
General	41.1 (8.05)		
Total	81.6 (14.6)		
**Cognition**			
Speed of Processing	29.4 (12.6)	46.6 (8.17)	<0.001^a^
Attention/Vigilance	31.7 (13.0)	48.9 (9.34)	<0.001^a^
Social Cognition	39.0 (10.4)	48.6 (10.0)	<0.001^a^
Working Memory	37.0 (10.8)	45.1 (9.09)	<0.001^a^
Reasoning/Problem Solving	39.6 (14.7)	47.7 (9.18)	<0.001^a^
Verbal Learning	34.4 (10.0)	40.4 (8.99)	<0.001^a^
Visual Learning	33.6 (9.88)	42.4 (9.92)	<0.001^a^
**SOD (U/ml)**	182 (18.7)	211 (28.7)	<0.001^a^

Data are expressed as mean ± SD.

*HC*, healthy controls; *SCZ*, schizophrenia; *TIV*, total intracranial volume; Smoking history defined as > 5 cigarettes/week for > 3 months; *PANSS*, positive and negative syndrome scale; *SOD*, superoxide dismutase.

^a^Two-sample t-test; ^b^Chi-square test.

### Symptom assessment and SOD laboratory analysis

After all subjects were enrolled, 5 ml of venous blood was collected from the elbow under fasting condition (12 h fasting) by full-time laboratory personnel in the morning of the next day from 6:30 a.m. to 7:30 a.m. to avoid the influence of biological rhythm changes of the measured factors. Venous blood samples were collected from the elbows into EDTA anticoagulant tubes at 4◦C for 10 min at 3000 rpm to separate the upper serum. SOD serum levels were measured by a colorimetry assay, using a Roche automatic biochemical analyzer (Roche Diagnostics, C8000, Germany).

### Magnetic resonance imaging acquisition parameters

T1-weighted MRI and rs-fMRI were acquired from a GE Discovery 750 3 T MRI scanner with an 8-channel phased array head coil. The T1 images were acquired with repetition time (TR) = 8.2 ms, echo time (TE) = 3.2 ms, field of view (FOV) = 256×256 mm2, slice number = 188, slice thickness = 1 mm, flip angle = 12° and 256×256 matrix. Rs-fMRI images were collected by using the echo planar imaging sequence (EPI) with TR = 2000 ms, TE = 30 ms, FOV = 220×220 mm2, slice number = 32, slice thickness = 4 mm, interslice gap = 0.5 mm, flip angle = 90°, and 64×64 matrix; the scanning time for each subject was approximately 6 min (resulting in 180 volumes). During scanning, participants were required to think about nothing, keep their heads still, and keep their eyes closed. All images were visually inspected to ensure that only images without visible artifacts were included in subsequent analyses.

### Gray matter volume analysis

Using the T1-weighted MRI data, we followed the standard pipeline of the CAT12 toolbox (http://www.neuro.uni-jena.de/cat) implemented in SPM12 to perform the VBM analysis. The main steps included affine normalization, nonlinear registration, bias field correction, tissue segmentation into gray matter, white matter and cerebrospinal fluid (CSF), spatial normalization into the Montreal Neurological Institute (MNI) space, resampling to 1.5×1.5×1.5 mm^3^ and nonlinear modulation [31, 32]. Finally, the obtained GM maps were smoothed using a 6-mm full-width at half maximum (FWHM) Gaussian kernel.

### fALFF analysis

The rs-fMRI data were preprocessed using SPM12 (https://www.fil.ion.ucl.ac.uk/spm/) and RESTplus V1.21 (http://www.restfmri.net). The processing steps were as follows. First, the initial ten volumes were removed, followed by slice-timing correction. Head motion less than 3 mm and 3° was corrected. Then, the time series of each subject was realigned by a linear transformation. After the realignment, the mean functional image was coregistered to the corresponding T1 image, which was further segmented into GM, white matter and CSF using a unified segment. Finally, the functional images were resampled to 3 × 3 × 3 mm^3^ and then normalized into the MNI space using the DARTEL technique. The data were subsequently smoothed spatially using a 6 mm FWHM Gaussian kernel. To calculate fALFF [27], the sum of the amplitude values in the low-frequency power range from 0.01 to 0.08 Hz was divided by the sum of the amplitudes over the entire detectable power spectrum (range: 0–0.25 Hz).

### Fusion analysis of multi-modal brain imaging data with reference

To ensure the two modalities had the same ranges, we firstly transform the 3D brain images from each participant into 1D vectors and compiled into a matrix for each of the two metrics: GMV, and fALFF (Nsubject × Nvoxel). Then, to ensure that both imaging modalities (GMV and fALFF) contributed equally to the fusion model and were not biased by their original numerical scales and units, we performed data normalization on each feature matrix. Specifically, for each metric’s matrix (Nsubject × Nvoxel), we normalized the data by using square root of mean of squared data for all subjects. This normalization procedure ensures that the total variance is comparable between the GMV and fALFF datasets, preventing either modality from disproportionately dominating the subsequent fusion analysis simply due to having larger numerical values. After that, two pre-processed MRI features were used for a guided fusion analysis of SOD levels in a multi-set canonical correlation analysis with reference plus joint independent component analysis (MCCAR + jICA) framework in the Fusion ICA Toolbox (FIT, http: //mialab.mrn.org/software/fit). MCCAR was proposed to simultaneously maximize the correlations of certain imaging components with the measure of interest, and inter-modality covariation. In the MCCAR model, the term containing the SOD reference signal (the term with λ) actively influences the decomposition process. It pushes the algorithm to find components in the neuroimaging data that not only covary across modalities but also have a strong linear relationship with SOD levels. As a result, it enables identification of a joint multimodal components that has robust correlations with both SOD and inter-modality correlations, which may not be detected by an unsupervised N-way multimodal fusion approach [25, 33]. The equation is shown below.

$$\max \mathop{\sum }\limits_{{\rm{k}},{\rm{j}}=1}^{{\rm{n}}}\left\{{\left\Vert {\rm{corr}}\left({{\bf{A}}}_{{\rm{k}}},{{\bf{A}}}_{{\rm{j}}}\right)\right\Vert }_{2}^{2}+2\lambda \cdot {\left\Vert {\rm{corr}}\left({{\bf{A}}}_{{\rm{k}}},{ref}\right)\right\Vert }_{2}^{2}\right\}$$

Where ref is an N × 1 vector, denoting the level of SOD, N is the subject number. Ak is a mixing matrix (subjects × number of components). corr(Ak, Aj) is the column-wise correlation between Ak and Aj, and corr(Ak, ref) is the column-wise correlation between Ak and ref. After optimization by MCCAR, we obtained a refined multimodal dataset that enhances components correlated with SOD and inter-modal covariation. The preprocessed data was then subjected to jICA analysis to extract spatially independent components (ICs). The jICA step ensures the independence of these spatial patterns, avoiding overlapping components and improving the biological interpretability of the results. Based on the minimum description length criteria, the IC number was estimated to be 9 and 2 for GMV and fALFF, respectively [34]. Nine ICs were chosen for further analysis as a slight overestimation of the IC number may improve decomposition accuracy in simulation [33–36]. A major problem in application of ICA is that the reliability of the estimated ICs is not known. ICASSO is a method for investigating the relations between estimates from Infomax ICA [37, 38]. The algorithmic and statistical reliability/stability is investigated by running the algorithm many times with different initial values or with differently bootstrapped data sets, respectively. The stability of each component cluster is quantified by the quality index (Iq index, range 0–1), and only components belonging to clusters with high stability (Iq ≥ 0.90) were retained for subsequent analysis [36, 39–41]. Components are defined by their spatial maps and their mixing coefficients. Finally, the primary variables of interest are mixing coefficients of each IC for each imaging measure. Two-sample t-tests were carried out on mixing coefficients of each component for each modality. We aimed to investigate the joint ICs, which are significantly correlated with SOD levels, covarying among modalities and discriminating between groups.

### Correlation with neurotransmitters

JuSpace is a spatial correlation analysis tool that links MRI data with neurotransmitter maps derived from nuclear imaging from a number of publicly available datasets [22, 30]. It offers a biologically meaningful framework that integrates neuroimaging with neurotransmitter information, providing insights into the biological underpinnings of brain function. Here, we utilized JuSpace (version 1.5) to perform spatial correlation analyses with PET and SPECT-derived maps of various neurotransmitter systems, including dopamine, serotonin, glutamate, NMDA, GABA, acetylcholine, opioid, cannabinoid, noradrenaline, and fluorodopa (Table S1 in Supplementary materials). These maps are derived from public studies using highly specific radiotracers (e.g., [¹¹C]Raclopride for D2 receptors, [¹¹C]DASB for SERT) and represent the non-displaceable binding potential, a measure of receptor availability and density. The spatial normalization of all maps to a standard template allows for voxel-wise spatial correlation analysis with our MRI-derived components. Components that showed significant differences in SOD-guided multimodal fusion analysis served as inputs for these correlations. We calculated Pearson correlation coefficients between the z-maps and neurotransmitter maps across the neuromorphometrics atlas. To determine statistical significance, we used spatial permutation-based null maps with 10000 permutations and applied Bonferroni correction for multiple testing adjustments (*p* value < 0.05).

### Statistical analysis

Statistical descriptive analyses of demographic and cognitive data were performed using the SPSS 25.0 software package (SPSS, Chicago, Illinois). The loading parameters for each component across modalities were compared using two-sample t-tests to identify the joint components that exhibited significant group differences and correlations with serum SOD levels. Multiple comparisons were corrected by the false discovery rate (FDR) method, with a corrected significance level of *p* < 0.05. To explore the impact of oxidative stress and neuroimaging features on cognitive function, we further investigated their associations using partial correlations adjusting for age, total intracranial volume (TIV), education, and gender.

## Results

### Serum levels of SOD and their relationship with clinical measurements

**Fig. 1:**
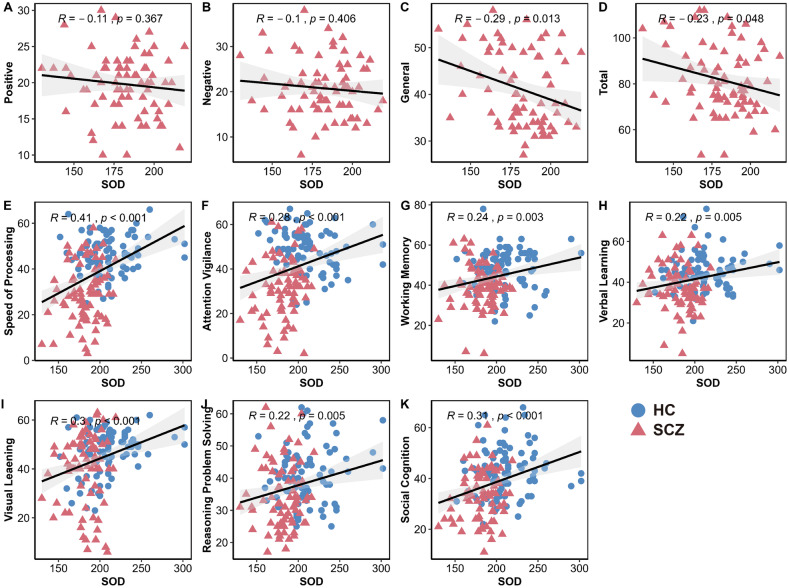
Correlations between serum levels of SOD and clinical measures. Negative correlations between serum levels of SOD and PANSS scores (**A**-**D**); Positive correlations between serum levels of SOD and cognitive function scores (**E**-**K**). Lower serum levels of SOD correspond to higher clinical symptom scores in general and total PANSS scores, suggesting more severe disability in schizophrenia. Higher serum levels of SOD correspond to higher cognitive function scores, indicating greater cognitive impairment in schizophrenia. Note that blue and red dots refer to HCs and SCZs, respectively. The gray regions in panels (A–K) indicate a 95% confidence interval.

Three SCZ patients and one HC were excluded for excessive head motion, yielding a final sample of 75 patients and 85 controls with usable neuroimaging data. The duration of the illness in the patient group was 7.2 ± 8.7 months. Serum levels of SOD were significantly reduced in SCZ patients (HC: 211 ± 28.7; SCZ: 182 ± 18.7; *p* < 0.001). Additionally, serum SOD levels are significantly negatively correlated with the general and total scores. Conversely, higher serum SOD levels are associated with higher cognitive function scores (degree of freedom, df = 158) (Fig. 1).

### Multimodal covarying imaging patterns associated with serum levels of SOD

**Fig. 2:**
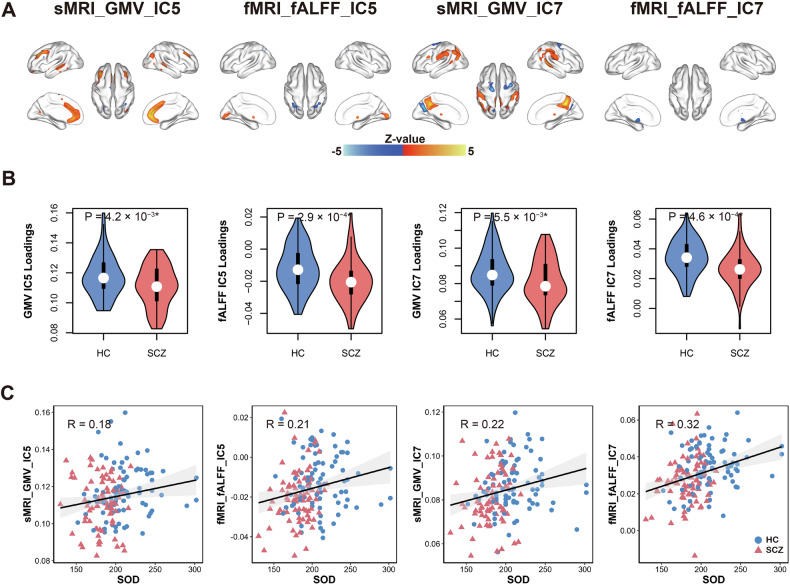
The IC5 and IC7 associated with serum levels of SOD show significant group difference in two modalities. Spatial maps visualized at |Z| > 2.5, in which the positive Z-scores (warm regions) mean HC subjects > SCZ patients and the negative Z-scores (cool regions) mean HC subjects < SCZ (**A**). Violinplot of the loading parameters of IC5 and IC7 that were adjusted as HC subjects > SCZ on the mean of loadings. Note that * means significance passed FDR corrected for multiple comparison (number of components * number of modalities) (**B**). Loadings of IC5/IC7 and SOD levels were positively correlated, thus SCZ corresponds to lower SOD serum levels and lower loading weights compared to HC subjects (**C**). Note that blue and red dots refer to HC and SCZ, respectively. The gray regions indicate a 95% confidence interval. sMRI, structural MRI; fMRI, functional MRI; GMV, grey matter volume; ∗ denotes FDR corrected for multiple comparison.

Two joint components (denoted as IC5 and IC7, with the same IC order in the two modalities) were both found to be significantly groups discriminatory (P_IC5_ = 0.004*, 0.0003*, P_IC7_ = 0.005*, 0.00005* for sMRI and functional MRI modalities, respectively, ∗ denotes FDR corrected for multiple comparison); and correlated with SOD (r = 0.18*, 0.21*, 0.22*, 0.32*, for sMRI and functional MRI modalities, respectively). The spatial maps were transformed into Z scores, visualized at |Z| > 2.5 as in Fig. 2A and all modalities of loading parameters (violinplot in Fig. 2B). The positive Z-scores (warm brain regions) indicate higher contribution in HC subjects than patients with SCZ and the negative Z-scores (cool brain regions) indicate higher contribution in patients with SCZ than HC subjects. The identified regions in IC5 and IC7 are summarized in Supplementary Table 2 for GMV and fALFF (Talairach labels). Figure 2C shows the correlations between IC5 and IC7 loadings and SOD levels (HC subjects: blue dots, SCZ: red dots); the higher IC loadings correspond to higher SOD levels in patients with SCZ. As shown in Fig. 2C, higher serum levels of SOD in SCZ are linked with increased values in both fALFF and grey matter in the anterior/posterior cingulate cortex (ACC/PCC) [Brodmann areas (BA) 10, 24, 25, 30, 32, 33], frontal cortex [BA 8, 9, 10], and angular gyrus [BA 39]. For functional MRI only, higher SOD levels are associated with lower fALFF in the cerebellar tonsil, culmen, and inferior parietal lobule. For grey matter only, reduced SOD associates with decreased GMV in the thalamus and lentiform nucleus.

### Partial correlation between independent component loadings (IC5/IC7) and clinical measurements

**Fig. 3:**
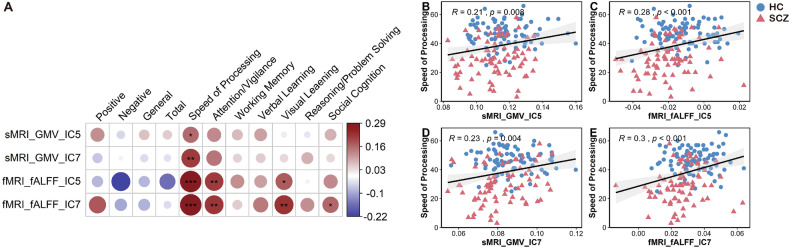
Correlations between loadings and clinical measures. **A** Heatmap shows no significant correlations between IC5/IC7 loadings and PANSS scores; Positive correlations between IC5/IC7 loadings in both structural and functional modalities and speed of processing, attention/vigilance, and visual learning. **B** Scatter plot of the sMRI_GMV_IC5 and speed of processing. **C** Scatter plot of the fMRI_fALFF_IC5 and speed of processing. **D** Scatter plot of the sMRI_GMV_IC7 and speed of processing. **E** Scatter plot of the fMRI_fALFF_IC7 and speed of processing. Pearson coefficients with p-values (*p < 0.05, **p < 0.01, and ***p < 0.001, respectively).

To examine the effects of oxidative stress and neuroimaging features on cognitive function, we further investigated their associations using partial correlations adjusting for age, TIV, education, and gender. Figure 3 shows correlations between IC5/IC7 loadings with PANSS and cognition measures. No significant correlations between IC5/IC7 loadings and PANSS scores. Positive correlations between IC5/IC7 loadings in both structural and functional modalities and speed of processing.

### Spatial correlations between neurotransmitters and the covariant structural-functional components associated with SOD levels

**Fig. 4:**
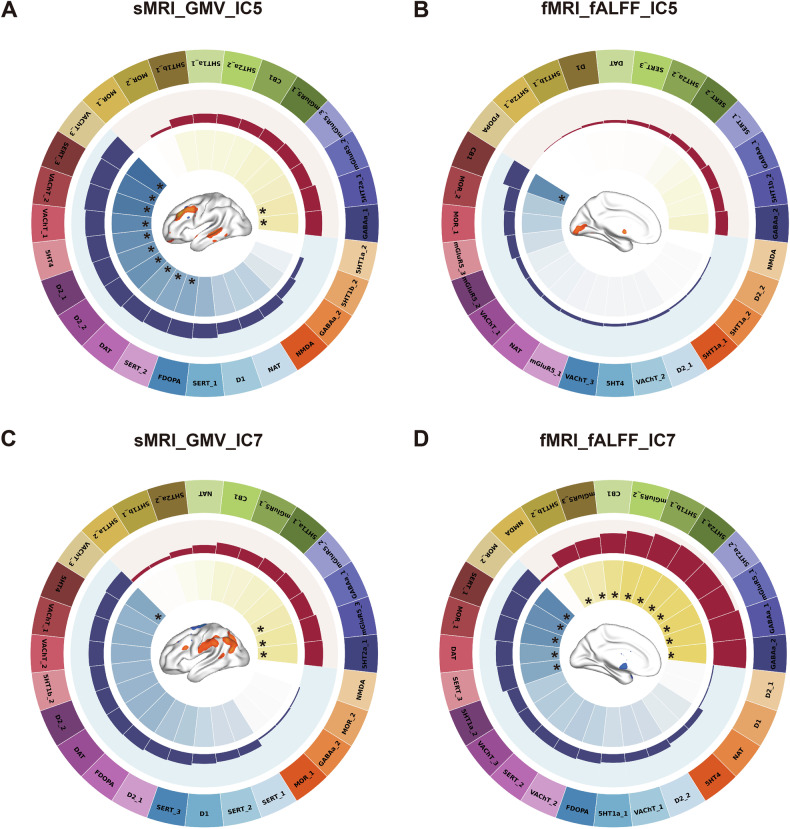
Spatial correlations between neurotransmitters and the covariant structural-functional components. **A** Correlations between GMV_IC5 and receptor systems. **B** Correlations between fALFF_IC5 and receptor systems. **C** Correlations between GMV_IC7 and receptor systems. **D** Correlations between fALFF_IC7 and receptor systems. The outermost ring displays the names and maps of 28 neurotransmitter receptors/ transporters. The second circle displays the cross-region Pearson correlation coefficients between these neurotransmitter maps and neural correlates of SOD; the light red background represents positive correlations, and the light blue background represents negative correlations. The innermost ring displays the permutation-based statistical significance of the spatial correlations, i.e., − log10(P); *p < 0.05, Bonferroni corrected. The z maps for the correlations between SOD and neuroimaging measures lie in the center. Abbreviations: fALFF, fractional amplitude of low-frequency fluctuations; GMV, gray matter volume; SOD, superoxide dismutase; 5-HT, 5-hydroxytryptamine; D, dopamine; GABA, gamma-aminobutyric acid; mGluR5, metabotropic glutamate 5; VAChT, vesicular acetylcholine transporter; CB1, cannabinoid type 1; DAT, dopamine transporter; FDOPA, fluorodopa; MOR, mu opioid receptor; NAT, noradrenaline transporter; SERT, serotonin transporter.

Cross-modal correlations showed associations between fALFF_IC5 and the cannabinoid system (CB1), as well as between fALFF_IC7 and the serotonergic system (5HT2a, 5HT1b, SERT DASB HC30 and SERT DASB HC100), the glutamatergic system (mGluR5_1, mGluR5_2 and mGluR5_3), μ-opioid receptor system and the GABAergic system (GABAa_FLUMAZENIL_HC11, GABAa_FLUMAZENIL_HC17). In addition, the cross-modal correlations showed negative associations between GMV_IC5 and the dopaminergic system (D2, DAT and FDOPA) as well as the cholinergic system (VAChT_feobv_HC4, VAChT_feobv_HC5 and VAChT_feobv_HC18). Furthermore, there are positive correlations between co-varying structural-functional patterns between IC7 and the serotonergic system (5HT2a), the GABAergic system (GABAa) and the glutamatergic system (mGluR5) (as shown in Fig. 4). Consequently, the covariant structural-functional components affected by SOD levels were associated with a higher spatial distribution of the serotonergic and GABAergic system, and a lower correlation with the distribution of the cholinergic and dopaminergic system in SCZ patients compared to HC. The detailed Fisher’s z scores are summarized in Supplementary Table 3.

## Discussion

**Fig. 5:**
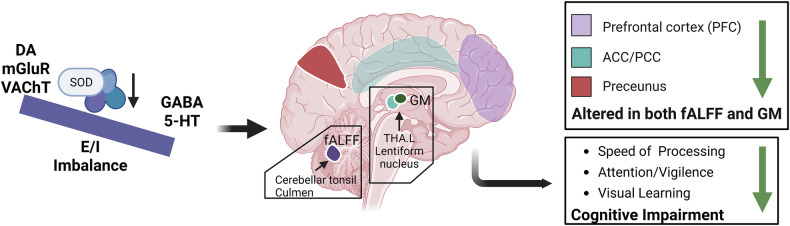
Summary of our findings about SOD dysregulation in SCZ. The redox imbalances caused by abnormal SOD levels affect the structure and function of important brain regions in SCZ patients, including a positive correlation with the distribution of serotonergic and GABAergic receptors, and a negative correlation with the distribution of cholinergic and dopaminergic receptors. Then, the structural-functional alteration of specific neurotransmitter systems caused by oxidative stress could explain part of the cognitive impairment of SCZ patients, especially the speed of processing. 5-HT, 5-hydroxytryptamine; DA, dopamine; GABA, gamma-aminobutyric acid; mGluR, metabotropic glutamate; VAChT, vesicular acetylcholine transporter; E/I, excitatory-inhibitory; fALFF, fractional amplitude of low frequency fluctuations; GM, gray matter; PFC, prefrontal cortex; ACC/PCC, anterior/posterior cingulate cortex; THA.L, left thalamus. (Created with bioRender.com).

In this study, we found a significant reduction in peripheral SOD levels in patients with first-episode drug-naïve SCZ, which was significantly negatively correlated with general and total pathological symptoms and cognitive function. We identified two multimodal neuroimaging patterns that are significantly influenced by changes in peripheral SOD levels. The brain regions involved in the multimodal imaging features were consistent with previous imaging studies of SCZ, especially in DMN key brain regions. Furthermore, the multimodal neuroimaging patterns influenced by peripheral SOD levels were significantly positively correlated with speed of processing. The multimodal neuroimaging features IC5/IC7 show significant spatial correlations with PET and SPECT-derived maps of various neurotransmitter systems. Consequently, the present results suggest that redox imbalances caused by abnormal SOD levels affect the structure and function of important brain regions in SCZ patients, including a positive correlation with the distribution of serotonergic and GABAergic receptors, and a negative correlation with the distribution of cholinergic and dopaminergic receptors (as shown in Fig. 5).

SCZ patients experience a decline in functions such as attention, memory, social cognition and speed of processing from early adulthood, with progressive deterioration throughout the course of the disease [42]. The convergent results showed cognitive impairment as measured by the MCCB in our study. Research suggests that brain networks composed of multiple regions are involved in the cognitive dysfunction of SCZ [43]. Notably, there is strong evidence that the DMN is most consistently associated with dysfunctional brain networks in SCZ [6, 44–47]. The DMN includes key regions such as the PCC, precuneus, medial prefrontal cortex, and bilateral temporoparietal junction [7]. The DMN is responsible for spontaneous perception and environmental monitoring and interacts antagonistically with the attention network [48, 49]. Consistent with the above studies, we found that altered structural-functional patterns influenced by peripheral SOD levels were concentrated in DMN-related networks. In addition, parvalbumin interneurons (PV-IN) in the prefrontal cortex and ACC have unique energy requirements, making them susceptible to the influence of various stressors [50].

Although the mechanisms of cognitive dysfunction in SCZ are not fully understood, oxidative stress is one of the neurochemical abnormalities [14, 51]. SOD, a crucial enzymatic antioxidant in the body, catalyzes the decomposition of superoxide anions into hydrogen peroxide (H2O2) and oxygen (O2), exhibiting its antioxidant properties. Several studies have shown increased oxidative products in patients with SCZ [12, 52–54]. Elevated levels of malondialdehyde (MDA), the final product of cellular lipid peroxidation, have been observed in the serum, red blood cells, and CSF of SCZ patients [53, 54]. The accumulation of oxidative products in the brain contributes to neurodegeneration, which is a pathological basis for brain aging and degeneration and is also a potential risk factor for cognitive impairment in SCZ patients [55–57]. Studies have confirmed that as oxidative stress increases, abilities such as language and visual learning decline significantly [58–60]. Fernandez A et al. found that re-expression of Txnrd2, a 22q11 gene essential for the degradation of reactive oxygen species in brain mitochondria (Txnrd2), can rescue impaired cognition associated with 22q11 deletion, further suggesting that oxidative stress could be involved in the process of cognitive impairment [59]. Furthermore, long-term treatment with NAC was found to be able to restore prefrontal hypomyelination and cognitive deficits in a SCZ rat model [60]. Therefore, restoring a state of redox homeostasis may be effective against cognitive deficits.

However, previous research findings are inconsistent regarding the role of SOD in patients with SCZ. Bai et al. found decreased serum SOD levels in patients with SCZ (n = 40 first-episode, drug-free and 40 chronically medicated patients), which is consistent with our results [61]. Consistently, CSF studies revealed 26.5–30% lower SOD1 in recent-onset SCZ, suggesting early CNS-specific redox disruption [62, 63]. Conversely, Jordan et al. observed a non-significant trend toward elevated serum SOD in 22 drug-naïve first-episode patients (p = 0.053) [52]. One explanation is that the antioxidant capacity of schizophrenia patients may go through various states over the durations of their illness. Due to the limitations of the study design, the current research cannot establish causality but rather provides evidence of association. Meanwhile, longitudinal studies are recommended in the future to further investigate the causal relationships between oxidative stress and schizophrenia. It is noteworthy that accumulating data indicate a close correlation between oxidative stress biomarkers in peripheral tissues and those in the CNS (CSF or post-mortem) [62–64]. Our results suggest that blood SOD levels may partially reflect the oxidative status of the CNS via the MCCAR + jICA framework.

A PET-derived study found that drug-naive SCZ patients exhibit reduced 5-HT2A receptor binding in the frontal cortex, which is negatively correlated with positive psychotic symptoms [65]. Glutamate is the primary excitatory neurotransmitter and its receptor dysfunction can lead to dopamine hyperactivity and influence the development of schizophrenia [2]. Additionally, the levels of dopamine and its receptors are elevated in certain brain regions of SCZ patients [66]. Dopamine signaling is associated with a concurrent decrease in whole brain functional connectivity and DMN activity. In contrast, 5-HT signaling is associated with increased DMN activity [67, 68]. In fact, the main excitatory and inhibitory neurotransmitters in the brain (dopamine, glutamate, GABA, and 5-HT) are crucial for maintaining a precise excitatory-inhibitory (E/I) balance [69, 70]. Coherently, we found significant associations between the altered structural-functional coupling observed in SCZ patients and the neurotransmitter receptor systems, which showed opposite correlation characteristics: negative correlations with the distribution of excitatory receptors and positive correlations with the distribution of inhibitory receptors. Therefore, we suggest that oxidative stress may be associated with an imbalance in the E/I ratio in the core brain regions of the DMN.

The validity of our spatial correlation analysis is supported by the use of the well-validated JuSpace toolbox and its curated repository of neurotransmitter maps derived from high-affinity radiotracers in large, independent cohorts [22, 30]. Furthermore, we employed spin-based permutation testing with FDR correction, which rigorously controls for spatial autocorrelation and multiple comparisons, ensuring that the reported correlations are unlikely to occur by chance. In addition, our spatial correlation analysis reveals shared topography between SOD-related brain component and specific neurotransmitter systems, but does not imply biological exclusivity. This reflects the biological reality of densely overlapping neurotransmitter systems in the human brain. Like the prefrontal cortex, which is a key region in our findings, contains high density of glutamate receptors alongside significant serotonergic and GABAergic inputs. Therefore, correlation with multiple systems likely indicates that SOD-related alterations occur within convergent hubs influenced by several neuromodulatory pathways. This systems-level perspective aligns with schizophrenia’s complex pathophysiology, which likely involves dysregulation across multiple chemical systems. While our approach cannot disentangle unique contributions of individual neurotransmitters, it successfully identifies a shared neuroanatomical architecture linking peripheral oxidative stress (SOD) to central neurochemistry, generating specific hypotheses for future investigation.

Our study has several limitations that should be noted. First, the predominance of early phase patients (70 percent ≤ 6 months) in our cohort enables detection of early redox dysregulation before compensatory adaptations obscure primary pathology. However, the dynamic neurobiology of schizophrenia requires longitudinal cohort research. To address this issue, large-scale multi-phase longitudinal cohorts should be established in the future. Second, our study focuses on a young cohort (mean age ~22 years) of first-episode, drug-naïve patients to specifically capture the early neuropathophysiology of schizophrenia. This design minimizes confounders from chronic illness, long-term treatment, and age-related comorbidities, allowing us to isolate associations that likely reflect core disease mechanisms proximate to illness onset, rather than downstream consequences of aging or chronicity. Therefore, our findings define the baseline pathological relationship between oxidative stress and brain integrity from which the illness evolves. While the question of how this relationship changes across the lifespan is vital, it is a subject for future longitudinal research; our study establishes the essential starting point for that next phase of inquiry. Third, despite the balanced smoking rates was observed in our study, residual confounding from light/unreported tobacco use cannot be excluded. Future study should prospectively collect smoking history in cohort designs across disease phases and use causal mediation models to quantify the specific contribution of tobacco to neuro-oxidative pathways. In addition, publicly available neurotransmitter receptor/transporter distribution maps and the JuSpace toolbox allow for cross-modal evaluation of neuroimaging data as well as molecular imaging profiles. However, the limited availability of specific radiotracers for key neurotransmitter systems (e.g., mGluR and NMDA receptors) constrained our ability to map neurochemical disturbances in a comprehensively way. While multimodal validation with PET/SPECT and postmortem atlases have partially mitigated this gap, further studies with novel tracers are required to fully address these limitations.

In summary, this study reveals the complex alterations in first-episode drug-naïve SCZ patients across structural, functional and neurochemical dimensions through multimodal analysis, highlighting the association between these changes and SOD-induced redox imbalances. Our study stands out for its innovative and data-driven approach, which minimizes potential bias from a priori hypotheses and strengthens the objectivity of our findings. A key innovation lies in our multimodal framework, which integrates structural, functional, and neurochemical data to provide a comprehensive and holistic understanding of brain abnormalities in SCZ patients, significantly enhancing the validity and reliability of our results. Another major strength is our focus on first-episode, drug-naive SCZ patients, a design choice that eliminates confounding effects of medication and chronic illness, thereby offering a clearer window into the early neurobiological mechanisms of SCZ. Most notably, we propose a novel and groundbreaking relationship between oxidative stress and the excitation/inhibition (E/I) ratio within the core regions of the DMN, shedding new light on the pathophysiology of SCZ and opening avenues for future research. While pharmacological interventions for SCZ can alleviate psychotic symptoms, they often fail to significantly improve social, cognitive, and occupational functioning [71–73]. Therefore, a structural-functional alteration of specific neurotransmitter systems caused by oxidative stress could explain part of the cognitive impairment of SCZ patients, especially the speed of processing. It provides valuable insights into the neurobiological basis of SCZ and could be an important target to restore cognitive deficits in speed of processing in individuals with SCZ.

## Supplementary information

Supplementary materials

## Data Availability

The datasets generated by this study are available from the corresponding authors upon request.

## Code availability

Computer code which was used for the analysis can be obtained from the authors upon reasonable request.
