# Supplementary material for: Multimodal neuroimaging reveals brain neurochemical disturbances associated with superoxide dismutase in first-episode drug-naïve schizophrenia
Source: Transl Psychiatry. 2026 Jan 5;16:19. doi: 10.1038/s41398-025-03801-w (PMC12804751; doi:10.1038/s41398-025-03801-w)
Supplement: Supplementary file 1 — Supplementary materials [file 41398_2025_3801_MOESM1_ESM.docx]

**Supplementary materials**

Supplementary materials for the article: “Multimodal neuroimaging reveals brain neurochemical disturbances associated with superoxide dismutase in first-episode drug-naïve schizophrenia”.

This file, Supplementary materials.doc contains three Supplementary Tables.

TableS1. Receptor/transporter maps

| **Neurotransmitter**  **receptor/transporter** | | **Tracer** | **Subjects** | **Source and reference** | |
| --- | --- | --- | --- | --- | --- |
| **Serotonin** | **5-HT1a_1** | [11C]WAY-100635 | 35 | [1] | |
|  | **5-HT1b_1** | [11C]P943 | 23 |  |  |
|  | **5-HT2a_1** | [18F]altanserin | 19 |  |  |
|  | **SERT_1** | [11C]DASB | 18 |  |  |
|  | **SERT_2** | [11C]MADAM | 10 | https://www.nitrc.org/projects/ki-5htt | |
|  | **SERT_3** | [11C]DASB | 100 | [2] | |
|  | **5-HT1a_2** | [11C]CUMI-101 | 8 |  |  |
|  | **5-HT1b_2** | [11C]AZ10419369 | 36 |  |  |
|  | **5-HT2a_2** | [11C]Cimbi-36 | 29 |  |  |
|  | **5-HT4** | [11C]SB207145 | 59 |  |  |
| **Glutamate** | **mGluR5_1** | [11C]ABP688 | 22 | PI: Pedro Rosa-Neto | |
|  | **mGluR5_2** | [11C]ABP688 | 28 | [3] | |
|  | **mGluR5_3** | [11C]ABP688 | 73 | [4] | |
| **Acetylcholine** | **VAChT_1** | [18 F] FEOBV | 18 | [5] | |
|  | **VAChT_2** | [18F]FEOBV | 4 | PI: Lauri Tuominen & Synthia Guimond | |
|  | **VAChT_3** | [18F]FEOBV | 5 | [6] | |
| **Opioid** | **MOR_1** | [11C]carfentanil | 204 | [7] | |
|  | **MOR_2** | [11C]carfentanil | 39 | [8] | |
| **Cannabinoid** | **CB1** | [11C]OMAR | 77 | [9] | |
| **Dopamine** | **D1** | [11C]SCH23390 | 13 | [10] | |
|  | **D2_1** | [11C]Raclopride | 7 | [11] | |
|  | **D2_2** | [11C]FLB-457 | 49 | [12] | |
|  | **DAT（SPECT）** | [123I]FP-CIT | 174 | [13] | |
|  | **FDOPA（SPECT）** | [123I]Ioflupane | 26 | [14] | |
| **Noradrenaline** | **NAT** | [11C]OMRB | 20 | [13] | |
| **NMDA** | **NMDA** | [18F]GE-179 | 29 | [15] | |
| **GABA** | **GABAa_1** | [11C]flumazenil | 6 | [16] |  |
|  | **GABAa_2** | [11C]flumazenil | 16 | [17] | |

Abbreviations: 5-HT, 5-hydroxytryptamine; CB1, cannabinoid type 1; D, dopamine; DAT, dopamine transporter; FDOPA, fluorodopa; GABAa, gamma-aminobutyric acid a; mGluR5, metabotropic glutamate type 5; MOR, mu opioid receptor; NAT, noradrenaline transporter; NMDA, N-methyl-D-aspartate; PET, positron emission tomography; SERT, serotonin transporter; SPECT, single photon emission computed tomography; VAChT, vesicular acetylcholine transporter.

TableS2. Anatomical information of the identified joint components in IC5 and IC7

| **sMRI_GM IC5 HC > SCZ** | **Brodmann Area** | **Vol(cc)L/R** | **random effects: Max Value (x, y, z)** |
| --- | --- | --- | --- |
| Anterior/Posterior Cingulate | 10, 24, 25, 30, 32, 33 | 8.6/9.4 | 7.1 (-3, 35, -4)/8.2 (4, 38, 2) |
| Sup/Med/Mid/Inf Frontal Gyrus | 6, 8, 9, 10, 11, 25, 46 | 9.9/5.3 | 6.5 (-36, 32, 27)/5.4 (36, 28, 33) |
| Sub-Gyral | - | 0.5/0.8 | 5.4 (-33, 32, 25)/5.5 (33, 36, 24) |
| Subcallosal Gyrus | 25 | 0.3/0.3 | 3.9 (-1, 18, -12)/5.2 (1, 21, -11) |
| Extra-Nuclear | - | 0.3/0.3 | 3.8 (-1, 23, 17)/5.0 (3, 24, 17) |
| Precentral Gyrus | 6, 9 | 1.1/0.3 | 4.9 (-37, 22, 35)/3.8 (37, 24, 35) |
| Superior/Mid/Inf Temporal Gyrus | 20, 21, 22, 37, 38, 39 | 4.9/3.2 | 4.8 (-52, -25, -4)/3.8 (50, -35, 5) |
| Angular Gyrus | 39 | -/0.8 | NA/3.9 (43, -67, 32) |
| Precuneus | 19, 39 | 0.1/0.5 | 2.7 (-16, -62, 18)/3.6 (40, -70, 35) |
| Lingual Gyrus | 17, 19 | -/0.6 | NA/3.4 (10, -93, 0) |
| Inferior Semi-Lunar Lobule | - | -/0.4 | NA/3.4 (39, -65, -41) |
| Cuneus | 17 | -/0.3 | NA/3.2 (12, -96, 2) |
| Middle/Inferior Occipital Gyrus | 18, 19 | -/0.5 | NA/2.9 (30, -90, 10) |
| **sMRI_GM IC5 HC < SCZ** |  |  |  |
| Lentiform Nucleus | - | 2.2/2.1 | 4.6 (-25, -3, 6)/4.7 (27, -3, 6) |
| Sub-Gyral | - | 1.1/0.4 | 4.3 (-37, 17, 23)/4.0 (34, -49, 38) |
| Extra-Nuclear | - | 0.8/1.2 | 3.5 (-30, -7, -1)/4.2 (31, -9, -2) |
| Inferior Parietal Lobule | 40 | -/0.4 | NA/3.6 (36, -52, 41) |
| Precuneus | - | 0.4/- | 3.5 (-24, -61, 36)/NA |
| Superior Temporal Gyrus | 41 | 0.4/- | 3.3 (-46, -24, 8)/NA |
| Superior/Med/Mid Frontal Gyrus | 6, 8 | 0.6/0.2 | 3.2 (-15, 23, 54)/2.7 (24, 32, 32) |
| Thalamus | - | 0.3/- | 2.9 (-12, -20, 12)/NA |
| **fMRI_fALFF IC5 HC > SCZ** |  |  |  |
| Superior/Mid/Med Frontal Gyrus | 6, 8, 9 | 1.7/0.7 | 3.6 (-15, 37, 51)/3.4 (27, 51, 36) |
| Cuneus | 17, 18, 23, 30 | 2.8/3.1 | 3.2 (-12, -78, 9)/3.4 (9, -84, 7) |
| Extra-Nuclear | - | 0.2/0.4 | 2.8 (-3, 0, 0)/3.2 (0, 0, -3) |
| Lingual Gyrus | 17, 18, 19 | 2.7/2.0 | 3.0 (-21, -58, 0)/3.2 (12, -84, 4) |
| Declive | - | 0.5/0.1 | 3.0 (-24, -83, -19)/2.9 (30, -83, -19) |
| Superior/Middle Temporal Gyrus | 21, 38 | -/0.5 | NA/3.0 (45, 2, -13) |
| Posterior Cingulate | 30, 31 | 0.4/0.4 | 2.6 (-12, -66, 14)/2.8 (21, -67, 9) |
| **fMRI_fALFF IC5 HC < SCZ** |  |  |  |
| Sub-Gyral | - | 1.4/1.2 | 3.6 (-30, -57, 33)/3.4 (30, -56, 39) |
| Precuneus | 7 | 1.3/0.6 | 3.6 (-21, -56, 39)/3.2 (24, -56, 42) |
| Superior/Inferior Parietal Lobule | 7, 39, 40 | 1.6/2.2 | 3.2 (-33, -59, 39)/3.2 (39, -48, 38) |
| Pyramis | - | -/0.3 | NA/3.0 (15, -77, -29) |
| Supramarginal Gyrus | 40 | 0.3/0.1 | 2.9 (-33, -51, 36)/2.7 (39, -45, 35) |
| **sMRI_GM IC7 HC > SCZ** |  |  |  |
| Posterior Cingulate/Cingulate Gyrus | 23, 24, 30, 31 | 5.3/3.6 | 7.3 (-7, -42, 37)/7.7 (9, -42, 37) |
| Precuneus | 7, 19, 31, 39 | 6.6/4.9 | 6.1 (-10, -45, 37)/7.0 (7, -46, 44) |
| Superior/Mid/ Inf Temporal Gyrus | 13, 21, 22, 39, 41, 42 | 2.9/5.9 | 4.6 (-40, -61, 24)/6.1 (43, -56, 24) |
| Postcentral/Precentral Gyrus | 2, 3, 4, 5, 40, 43 | 3.4/4.3 | 5.1 (-49, -23, 36)/4.2 (46, -23, 38) |
| Superior/Inferior Parietal Lobule | 2, 7, 39, 40 | 5.2/4.7 | 4.4 (-46, -55, 40)/5.0 (36, -37, 41) |
| Sub-Gyral | - | 1.6/1.5 | 4.8 (-39, 19, 23)/4.5 (46, -35, 3) |
| Thalamus | - | 1.7/1.2 | 4.7 (-10, -21, 11)/3.8 (12, -21, 9) |
| Pyramis | - | 0.8/0.4 | 4.2 (-33, -69, -31)/4.0 (36, -67, -31) |
| Paracentral Lobule | 5 | 0.6/0.3 | 4.0 (-1, -41, 48)/4.2 (6, -45, 58) |
| Tuber | - | 1.1/0.8 | 4.2 (-30, -71, -29)/4.1 (33, -70, -29) |
| Supramarginal Gyrus | 40 | 0.8/0.7 | 3.8 (-49, -53, 37)/4.1 (43, -51, 26) |
| Cerebellar Tonsil | - | 0.7/0.4 | 4.0 (-40, -63, -31)/3.7 (37, -63, -32) |
| Angular Gyrus | 39 | 1.1/0.1 | 4.0 (-46, -56, 36)/2.6 (42, -58, 31) |
| Insula | 13 | -/0.6 | NA/3.9 (49, -40, 19) |
| Superior/Middle Frontal Gyrus | 46 | 0.4/- | 3.4 (-42, 19, 25)/NA |
| Uvula | - | 0.4/0.3 | 3.2 (-28, -77, -25)/3.4 (30, -76, -25) |
| **sMRI_GM IC7 HC < SCZ** |  |  |  |
| Precuneus/Cuneus | 7, 31 | 3.0/0.7 | 5.5 (-13, -61, 29)/4.1 (15, -58, 33) |
| Superior/Middle/Med Frontal Gyrus | 6 | 3.9/5.7 | 5.1 (-16, -57, 22)/3.8 (24, -5, 55) |
| **fMRI_fALFF IC7 HC < SCZ** |  |  |  |
| Cerebellar Tonsil | - | 0.8/0.9 | 3.8 (-12, -43, -41)/4.1 (12, -43, -41) |
| Culmen | - | 1.3/1.6 | 3.7 (-15, -30, -16)/3.8 (15, -30, -16) |
| Superior Temporal Gyrus | 38 | 0.6/0.7 | 3.4 (-39, 8, -21)/3.2 (39, 11, -21) |

TableS1 listed the identified regions (z > 2.5), GMV and fALFF (Talairach labels). For each hemisphere (L = left; R = right), the maximum z-value and MNI coordinate are provided. The volume of voxels in each area is provided in cubic centimetres (cc); the table displays clusters > 0.2 cc.

TableS3. Associations between covariant structural-functional components and neurotransmitter activity maps

| **PET/SPECT map** | **Fisher`s z (Pearson r)** | | | | ***p* (parametric)** | | | |
| --- | --- | --- | --- | --- | --- | --- | --- | --- |
| **Components** | GMV_IC5 | fALFF_IC5 | GMV_IC7 | fALFF_IC7 | GMV_IC5 | fALFF_IC5 | GMV_IC7 | fALFF_IC7 |
| **5-HT1a_1** | 0.18 | -0.04 | 0.25 | -0.17 | 4.91E-02 | 6.95E-01 | 7.98E-03 | 7.15E-02 |
| **5-HT1b_1** | 0.17 | 0.03 | 0.07 | 0.51 | 6.77E-02 | 7.26E-01 | 4.79E-01 | **6.11E-08*** |
| **5-HT2a_1** | 0.31 | 0.02 | 0.35 | 0.53 | **8.73E-04*** | 8.66E-01 | **2.37E-04*** | **2.21E-08*** |
| **SERT_1** | -0.28 | 0.15 | -0.16 | -0.36 | 2.98E-03 | 1.11E-01 | 7.76E-02 | **1.37E-04*** |
| **SERT_2** | -0.30 | 0.12 | -0.17 | -0.22 | **1.17E-03*** | 2.04E-01 | 7.30E-02 | 2.10E-02 |
| **SERT_3** | -0.41 | 0.08 | -0.23 | -0.31 | **1.67E-05*** | 3.86E-01 | 1.42E-02 | **1.03E-03*** |
| **5-HT1a_2** | -0.01 | -0.04 | 0.07 | -0.23 | 9.00E-01 | 6.98E-01 | 4.66E-01 | 1.49E-02 |
| **5-HT1b_2** | -0.09 | 0.19 | -0.28 | 0.30 | 3.44E-01 | 4.03E-02 | 3.00E-03 | **1.21E-03*** |
| **5-HT2a_2** | 0.19 | 0.09 | 0.12 | 0.54 | 4.61E-02 | 3.47E-01 | 1.81E-01 | **1.45E-08*** |
| **5-HT4** | -0.36 | -0.06 | -0.29 | -0.12 | **1.28E-04*** | 5.26E-01 | 2.14E-03 | 2.09E-01 |
| **mGluR5_1** | 0.23 | -0.07 | 0.22 | 0.55 | 1.32E-02 | 4.53E-01 | 1.74E-02 | **8.90E-09*** |
| **mGluR5_2** | 0.26 | -0.10 | 0.28 | 0.44 | 5.30E-03 | 2.87E-01 | 3.03E-03 | **3.45E-06*** |
| **mGluR5_3** | 0.25 | -0.17 | 0.34 | 0.32 | 8.39E-03 | 7.21E-02 | **3.41E-04*** | **7.33E-04*** |
| **VAChT_1** | -0.37 | -0.09 | -0.29 | -0.17 | **6.88E-05*** | 3.38E-01 | 1.79E-03 | 6.28E-02 |
| **VAChT_2** | -0.40 | -0.06 | -0.29 | -0.22 | **1.80E-05*** | 4.99E-01 | 1.96E-03 | 1.73E-02 |
| **VAChT_3** | -0.43 | -0.07 | -0.31 | -0.23 | **5.69E-06*** | 4.83E-01 | **9.15E-04*** | 1.49E-02 |
| **MOR_1** | 0.01 | -0.21 | -0.04 | -0.36 | 9.02E-01 | 2.10E-02 | 6.54E-01 | **1.49E-04*** |
| **MOR_2** | 0.06 | -0.23 | -0.03 | -0.37 | 5.35E-01 | 1.41E-02 | 7.84E-01 | **6.97E-05*** |
| **CB1** | 0.20 | -0.35 | 0.22 | 0.38 | 3.07E-02 | **8.84E-05*** | 1.96E-02 | **5.47E-05*** |
| **D1** | -0.22 | 0.05 | -0.19 | -0.03 | 1.64E-02 | 6.26E-01 | 4.04E-02 | 7.30E-01 |
| **D2_1** | -0.35 | -0.06 | -0.24 | -0.03 | **1.95E-04*** | 4.85E-01 | 9.12E-03 | 7.10E-01 |
| **D2_2** | -0.35 | -0.03 | -0.28 | -0.17 | **1.71E-04*** | 7.15E-01 | 2.80E-03 | 7.23E-02 |
| **DAT**  **(SPECT)** | -0.32 | 0.06 | -0.28 | -0.32 | **6.92E-04*** | 5.04E-01 | 3.11E-03 | **7.57E-04*** |
| **FDOPA**  **(SPECT)** | -0.30 | 0.01 | -0.27 | -0.20 | **1.19E-03*** | 9.47E-01 | 3.65E-03 | 2.88E-02 |
| **NAT** | -0.22 | -0.08 | 0.16 | -0.12 | 2.10E-02 | 3.63E-01 | 9.21E-02 | 1.82E-01 |
| **NMDA** | -0.21 | 0 | 0 | 0.06 | 2.72E-02 | 9.75E-01 | 9.72E-01 | 5.10E-01 |
| **GABAa_1** | 0.32 | 0.19 | 0.30 | 0.61 | **6.72E-04*** | 4.03E-02 | **1.15E-03*** | **2.60E-10*** |
| **GABAa_2** | -0.14 | 0.24 | -0.04 | 0.82 | 1.40E-01 | 9.75E-03 | 7.02E-01 | **0.00E+00*** |

Abbreviations: 5-HT, 5-hydroxytryptamine; CB1, cannabinoid type 1; D, dopamine; DAT, dopamine transporter; FDOPA, fluorodopa; GABAa, gamma-aminobutyric acid a; mGluR5, metabotropic glutamate type 5; MOR, mu opioid receptor; NAT, noradrenaline transporter; NMDA, N-methyl-D-aspartate; PET, positron emission tomography; SERT, serotonin transporter; SPECT, single photon emission computed tomography; VAChT, vesicular acetylcholine transporter. **p* < 0.05, Bonferroni corrected.

**REFERENCES**

1. Savli M, Bauer A, Mitterhauser M, Ding Y-S, Hahn A, Kroll T *et al.* Normative database of the serotonergic system in healthy subjects using multi-tracer PET*.* *NeuroImage* 2012;63:447–459.

2. Beliveau V, Ganz M, Feng L, Ozenne B, Højgaard L, Fisher PM *et al.* A high-resolution in vivo atlas of the human brain's serotonin system*.* *J Neurosci* 2017;37:120–128.

3. DuBois JM, Rousset OG, Rowley J, Porras-Betancourt M, Reader AJ, Labbe A *et al.* Characterization of age/sex and the regional distribution of mGluR5 availability in the healthy human brain measured by high-resolution [(11)C]ABP688 PET*.* *Eur J Nucl Med Mol Imaging* 2016;43:152–162.

4. Smart K, Cox SML, Scala SG, Tippler M, Jaworska N, Boivin M *et al.* Sex differences in [11C]ABP688 binding: a positron emission tomography study of mGlu5 receptors*.* *Eur J Nucl Med Mol Imaging* 2019;46:1179–1183.

5. Aghourian M, Legault-Denis C, Soucy JP, Rosa-Neto P, Gauthier S, Kostikov A *et al.* Quantification of brain cholinergic denervation in Alzheimer's disease using PET imaging with [18F]-FEOBV*.* *Mol Psychiatry* 2017;22:1531–1538.

6. Bedard M-A, Aghourian M, Legault-Denis C, Postuma RB, Soucy J-P, Gagnon J-F *et al.* Brain cholinergic alterations in idiopathic REM sleep behaviour disorder: a PET imaging study with 18F-FEOBV*.* *Sleep Med* 2019;58:35–41.

7. Kantonen T, Karjalainen T, Isojärvi J, Nuutila P, Tuisku J, Rinne J *et al.* Interindividual variability and lateralization of μ-opioid receptors in the human brain*.* *NeuroImage* 2020;217:116922.

8. Turtonen O, Saarinen A, Nummenmaa L, Tuominen L, Tikka M, Armio R-L *et al.* Adult attachment system links with brain mu opioid receptor availability in vivo*.* *Biol Psychiatry Cogn Neurosci Neuroimaging* 2021;6:360–369.

9. Normandin MD, Zheng M-Q, Lin K-S, Mason NS, Lin S-F, Ropchan J *et al.* Imaging the cannabinoid CB1 receptor in humans with [11C]OMAR: assessment of kinetic analysis methods, test-retest reproducibility, and gender differences*.* *J Cereb Blood Flow Metab* 2015;35:1313–1322.

10. Kaller S, Rullmann M, Patt M, Becker G-A, Luthardt J, Girbardt J *et al.* Test-retest measurements of dopamine D1-type receptors using simultaneous PET/MRI imaging*.* *Eur J Nucl Med Mol Imaging* 2017;44:1025–1032.

11. Alakurtti K, Johansson JJ, Joutsa J, Laine M, Bäckman L, Nyberg L *et al.* Long-term test-retest reliability of striatal and extrastriatal dopamine D2/3 receptor binding: study with [(11)C]raclopride and high-resolution PET*.* *J Cereb Blood Flow Metab* 2015;35:1199–1205.

12. Zakiniaeiz Y, Hillmer AT, Matuskey D, Nabulsi N, Ropchan J, Mazure CM *et al.* Sex differences in amphetamine-induced dopamine release in the dorsolateral prefrontal cortex of tobacco smokers*.* *Neuropsychopharmacology* 2019;44:2205–2211.

13. Hesse S, Becker G-A, Rullmann M, Bresch A, Luthardt J, Hankir MK *et al.* Central noradrenaline transporter availability in highly obese, non-depressed individuals*.* *Eur J Nucl Med Mol Imaging* 2017;44:1056–1064.

14. García-Gómez FJ, García-Solís D, Luis-Simón FJ, Marín-Oyaga VA, Carrillo F, Mir P *et al.* [Elaboration of the SPM template for the standardization of SPECT images with 123I-Ioflupane]*.* *Rev Esp Med Nucl Imagen Mol* 2013;32:350–356.

15. Galovic M, Al-Diwani A, Vivekananda U, Torrealdea F, Erlandsson K, Fryer TD *et al.* In vivo NMDA receptor function in people with NMDA receptor antibody encephalitis*.* *medRxiv* 2021:2021.12.04.21267226.

16. Dukart J, Holiga Š, Chatham C, Hawkins P, Forsyth A, McMillan R *et al.* Cerebral blood flow predicts differential neurotransmitter activity*.* *Sci Rep* 2018;8:4074.

17. Nørgaard M, Beliveau V, Ganz M, Svarer C, Pinborg LH, Keller SH *et al.* A high-resolution in vivo atlas of the human brain's benzodiazepine binding site of GABA_A_ receptors*.* *NeuroImage* 2021;232:117878.
